# Treatment and Outcomes of Chronic Locked Posterior Shoulder Dislocations: A Retrospective Case Series

**DOI:** 10.3390/jcm14248955

**Published:** 2025-12-18

**Authors:** Marco Filipponi, Alberto Casto, Giuseppe Rollo, Filippo Tonelli, Andrea Pautasso, Fabio D’Angelo, Pietro Maniscalco, Corrado Ciatti, Paolo Pichierri

**Affiliations:** 1 Department of Orthopaedics and Traumatology, Vito Fazzi Hospital, 73100 Lecce, Italy; filipponimarco@yahoo.it (M.F.);; 2 Department of Orthopedic Surgery, Santa Maria Annunziata Hospital, ASL Toscana Centro—Via Antella 58, 50012 Bagno a Ripoli, Italy; 3 Orthopaedic and Traumatology Department, Circolo Hospital and Macchi Foundation in Varese—University Hospital, ASST Sette Laghi, Viale Luigi Borri, 55, 21100 Varese, Italy; 4 Department of Biotechnology and Life Sciences (DBSV), University of Studies Insubria, Via J.H. Dunant, 3, 21100 Varese, Italy; 5 Department of Medicine and Aging Sciences, University of Chieti-Pescara, 66100 Chieti, Italy; 6 Department of Medicine and Surgery, University of Parma, 43126 Parma, Italy; 7 Department of Orthopedics and Traumatology, Guglielmo da Saliceto Hospital, Via Taverna 49, 29121 Piacenza, Italy; 8 Ospedale Francesco Ferrari—Casarano, 73042 Casarano, Italy

**Keywords:** chronic posterior shoulder dislocations, McLaughlin lesion, subscapularis transfer

## Abstract

**Background/Objectives**: Chronic locked posterior shoulder dislocations (PSDs) are rare and often misdiagnosed, leading to delayed treatment and complex management. This study retrospectively evaluates surgical outcomes in patients treated for chronic PSDs and reports our clinical experience. **Methods**: Ten male patients with chronic PSDs treated between 2016 and 2022 at “Vito Fazzi Hospital” (Lecce) were analyzed. Lesions were classified according to the Randelli system (Type 1: 20–50% bone loss; Type 2: >50%; Type 3: fracture dislocation without bone loss; Type 4: multifragmentary fracture dislocation). Surgical options—subscapularis transposition, bone grafting, osteosynthesis, and reverse shoulder arthroplasty—were selected based on lesion type, age, and functional demand. Follow-ups at 1, 3, 6, and 12 months assessed ROM, SF-36, and SDQ scores. **Results**: Six patients had Type 1 lesions, two Type 2, and two Type 4. The mean diagnostic delay was 6 weeks (up to 5 months). Early follow-ups showed superior ROM and SDQ in patients with reverse prostheses, while at 12 months, cancellous grafts achieved better functional recovery. Subscapularis transpositions resulted in minor internal rotation loss and increased pain. One Type 4 case developed avascular necrosis. Mean healing time was 2.9 ± 0.5 months. Although SDQ differences at 12 months were not significant, internal rotation was reduced by 10% in patients treated with the McLaughlin technique (*p* < 0.05). **Conclusions**: Prompt diagnosis and tailored surgical management are key to favorable outcomes in chronic PSDs. While various techniques provide good results, subscapularis transposition should be limited to unstable cases, and osteosynthesis should be used only when strictly indicated due to necrosis risk.

## 1. Introduction

The glenohumeral joint provides the widest range of motion in the human body, but this mobility comes at the expense of intrinsic stability. As a result, anterior dislocations predominate (95%), whereas posterior shoulder dislocations (PSDs) remain rare (4%) and frequently under-recognized [1,2]. PSDs typically arise from high-energy trauma or violent muscle contractions during seizures or electrical shock, but may also progress insidiously through repetitive microtrauma leading to chronic posterior instability.

A critical clinical issue is that unrecognized or inadequately reduced acute PSDs may rapidly evolve into a chronic locked dislocation. This transition is facilitated by well-documented diagnostic pitfalls: pain-limited radiographs that fail to capture appropriate trauma series views, limited access to advanced imaging such as CT in emergency settings, and a low index of suspicion because posterior dislocations are far less common than anterior ones. Consequently, between 50% and 79% of PSDs are initially overlooked [2,3], and many are diagnosed only after several weeks, effectively meeting criteria for chronicity. Reported delays average up to 5.8 months [4], a figure reflected in our cohort, where mean diagnostic delay was six weeks, with extremes of up to five months.

Associated injuries are common. Rouleau et al. reported reverse Hill-Sachs lesions (rHSL) in 29% of cases, followed by humeral neck fractures (18.5%), lesser tuberosity (14.3%), greater tuberosity (7.8%), and other periarticular fractures (6%) [5,6]. The McLaughlin lesion represents the characteristic anterolateral humeral head impaction often combined with posterior glenoid or soft-tissue lesions.

Although frequently overlooked, PSDs demonstrate characteristic radiographic signs such as the “light bulb” and “edge” signs [7]. Computed tomography is essential to quantify the rHSL, evaluate associated fractures, and plan surgery. Locked posterior dislocations are defined by irreducibility in the presence of an rHSL.

**Figure 1:**
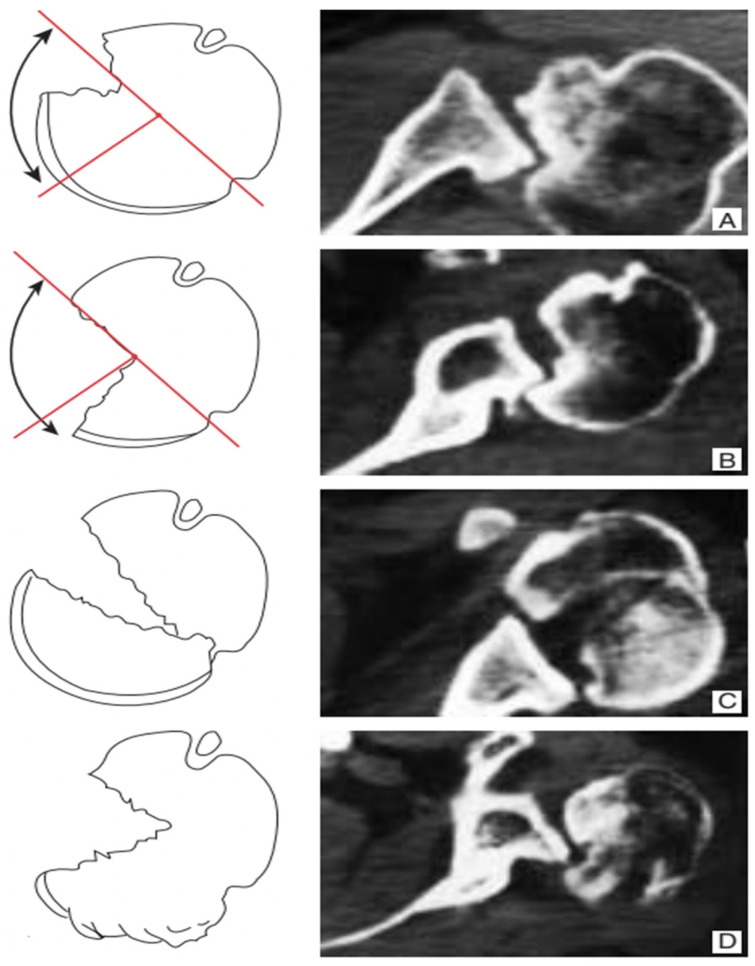
Classification of chronic posterior shoulder dislocation and fracture dislocation. Type 1 (**A**) humeral bone deficiency extending from 20 to 50% of the articular surface of the humeral head; Type 2 (**B**) humeral bone deficiency extending to more than 50% of the articular surface of the humeral head; Type 3 (**C**) fracture dislocation of the humeral head in the absence of bone deficiency. Type 4 (**D**) fracture dislocation with multifragmentation of the humeral head.

Several classification systems exist [8,9]. The Randelli classification is particularly useful because it integrates the extent of humeral head bone loss, dislocation direction, and the presence of a posterior fracture rim, enabling rational selection of surgical strategies based on radiographs and CT—including 3D reconstructions. It distinguishes four types of chronic posterior fracture dislocation:

We therefore have the following four types of confirmed posterior fracture dislocation (Figure 1):

- Type 1: chronic posterior dislocation is associated with humeral bone deficit ranging from 20% to 50% of the extension of the articular surface; it is the most frequent type;
- Type 2: the deficit is greater than 50%;
- Type 3: fracture dislocation of the humeral head, in which the continuity between the humeral head and the diaphysis is ensured by a posterior bony portion which acts as a hinge between the two structures. The fracture can be localized at the anatomical neck level, or it can involve a medial portion of the diaphysis. The articular pro-file of the humeral head is preserved.
- Type 4: the fracture is multi-fragmentary with complete subversion of the profile of the articular surface [4].

Surgical management for chronic PSDs is highly variable, dictated by factors such as humeral defect size, chronicity, and presence of osteoarthritis. Treatment options range from conservative approaches for minor defects to complex surgical interventions. For smaller reverse Hill-Sachs lesions (up to 40%), techniques like subscapularis tendon transposition (McLaughlin procedure) or lesser tuberosity transposition are employed. Larger defects (around 50%) may be addressed with bone grafting, while extensive bone loss (Type 2) or multifragmentary fracture dislocations (Type 4) often necessitate shoulder replacement. Type 3 lesions typically require open reduction and osteosynthesis, though with a recognized risk of avascular necrosis [10,11,12].

The treatment algorithm described by Aydin et al. recommends managing articular surface defects up to 25% with closed reduction in recent-onset cases, or open reduction when the dislocation is irreducible or stability cannot be maintained. If the joint remains stable after reduction, no additional intervention on the posterior capsulolabral complex is required; persistent instability, however, warrants open reduction and posterior capsulolabral reconstruction with posterior bone block augmentation fixed with cannulated screws [6,7].

For reverse Hill-Sachs lesions involving 25–40% of the articular surface, both subscapularis tendon transposition (McLaughlin procedure) and lesser tuberosity transfer (Hawkins modification) represent valid options. In chronic cases with defects approaching 50% and preserved bone stock, reconstruction with cancellous bone grafting may prevent the need for arthroplasty [8]. When the humeral head deficit exceeds 50% (Type 2), shoulder replacement—or, in selected low-demand patients, nonoperative management—should be considered according to age and functional expectations. Type 3 lesions require open reduction and internal fixation, with appropriate counseling about the risk of avascular necrosis. Type 4 fractures, characterized by complete articular disruption, invariably necessitate hemiarthroplasty or total shoulder arthroplasty.

Given the diagnostic and therapeutic complexities of chronic locked posterior shoulder dislocations, the primary aim of this study was to retrospectively evaluate the surgical outcomes of patients treated at the ‘Vito Fazzi Hospital’ of Lecce between 2016 and 2022. Specifically, our objective is to present our case series of patients affected by this rare pathology, highlighting the individualized surgical treatment approaches adopted in accordance with the Randelli classification. Our secondary goal was to share our clinical experience, contributing to the understanding of optimal management strategies for this challenging condition.

## 2. Materials and Methods

We performed a retrospective study including 10 patients with chronic shoulder dislocation fracture which were missed in the acute phase. Patients were assessed in our institution between 2016 and 2022. This is a retrospective study conducted at Orthopedics and Traumatology UOC of the Vito Fazzi Hospital in Lecce (ASL Lecce). During the specified period, a total of 20 patients with chronic locked posterior shoulder dislocations were treated. Of these, 10 patients were excluded. Specifically, 4 patients were excluded due to an acute diagnosis (within 3 weeks of trauma), and 6 patients were excluded due to severe comorbidities (e.g., severe neurological disorders, advanced degenerative joint disease unrelated to the PSD, or active systemic infections) that could significantly influence the outcomes. The remaining 10, who met all inclusion criteria, were included in the analysis.

The time lapse between the trauma and the diagnosis of chronic posterior shoulder fracture dislocation varied from a minimum of 3 weeks to a maximum of 5 months.

The case series collected consists of the following patients (Table 1):

- A total of 6 patients with type 1 lesion, of which 5 on the right side and 1 on the left side, with an average age of 38.5 years with a range from 25 to 47 years;
- A total of 2 patients with type 2 lesion, of which 1 on the right side and 1 on the left side, with an average age of 54.5 years with a range from 49 to 58 years;
- A total of 2 patients with type 4 lesion, of which 1 on the right side and 1 on the left side, with an average age of 43.5 years with a range from 41 to 46 years.

**Table 1:** Demographic and lesion characteristics of the study cohort.

PATIENTS	AGE	GENDER	SIDE	AGE	TIMING	SURGERY
6 Type 1	38.5 (25–47)	4 M 0 F	5 R 1 L	38.5 y (25–47)	2 patients 3 weeks 2 patients 6–8 weeks 2 patients 4 weeks	2 wads 2 transpositions 2 grafts
2 Type 2	54.25 (49–58)	4 M 0 F	1 R 1 L	54.25 y (49–58)	2 patients 8 weeks	2 reverse
2 Type 4	43.5 (41–46)	2 M 0 F	1 R 1 L	43.5 y (41–46)	3 weeks	1 osteosynthesis 1 reverse

The surgical procedures and subsequent follow-ups of the patients (at 1, 3, 6 and 12 months post-operative) were performed by the two authors (M.F. and A.C.). When the patient was unable to attend the visit, they were contacted by telephone.

The definitive diagnosis of the posterior shoulder fracture-dislocation status was performed via CT; which to evaluate the type and extent of involvement of the humeral head. MRI was used to assess soft tissues, to rule out labrum or the rotator cuff associated pathology

We excluded patients with lesions that involved the posterior glenoid wall, a diaphyseal extension of the fracture or neuro-vascular lesions were excluded from this study, as were those in whom the diagnosis was made acutely. Patients with severe comorbidities were also excluded from the study

The size of the reverse Hill-Sachs defect was measured and expressed as a percentage of the total articular surface, in order to classify the lesion according to the 4 aforementioned groups.

### Surgical Procedures

All patients underwent surgery under general anesthesia in a beach-chair position via a delto-pectoral approach. Reduction in the glenoid joint was the initial step. The specific surgical approach was individualized based on the Randelli classification, patient age, and functional demands. We selected the Randelli classification because it directly correlates lesion size, chronicity, and reconstructive options, allowing consistent decision-making across heterogeneous cases.

**Figure 2:**
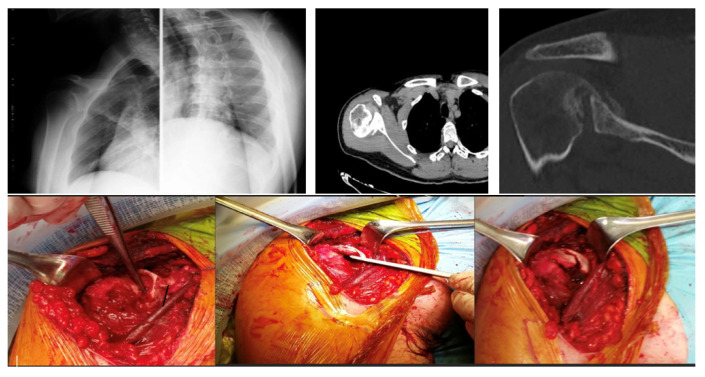
A 28-year-old man diagnosed with type 1 injury 3 weeks after the trauma treated with reduction in the dislocation, detachment of the subscapularis, reduction in the articular surface, bore with spongy bank bone and suture in anatomical position with transosseous stitches of the tendon.

**Figure 3:**
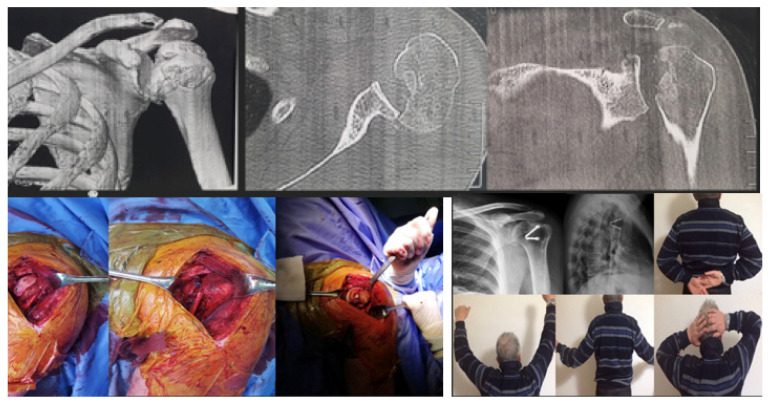
A 54-year-old man with type 1 injury 4 weeks after trauma. X-ray and clinical check-up at 12 months.

**Figure 4:**
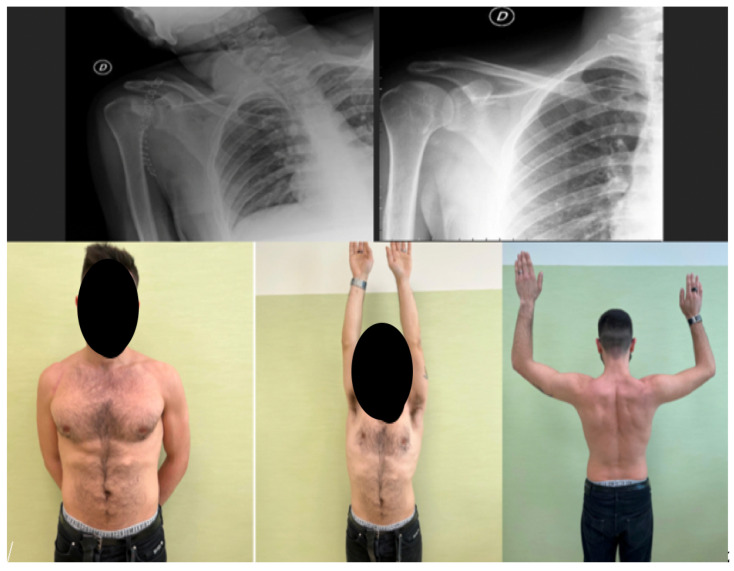
A 47-year-old man with type 1 injury 6 weeks after trauma; we proceeded with reduction in the dislocation, bleeding of the bottom of the lesion and transposition of the subscapularis tendon anchored to the bottom with transosseous stitches.

**Figure 5:**
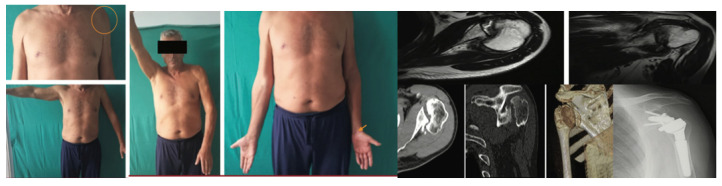
A 58 year old man with type 2 lesion for approximately 5 months. The circle shows the slight morphological alteration of the shoulder. Reverse prosthesis operation.

- Bone Grafting (Randelli Type 1): For Type 1 lesions with repairable impact injuries, particularly in younger patients and within a few weeks of trauma, bone grafting was performed. This involved detaching the subscapularis tendon, elevating the articular surface, and filling the defect. Bone grafting was specifically employed in cases classified as Randelli Type 1, characterized by a bone loss ranging from 20% to 50% of the humeral head articular surface. In all such instances, femoral head allografts sourced from a tissue bank were utilized. These allografts underwent precise intraoperative shaping to achieve an accurate press-fit within the humeral head defect. Fixation was then achieved using cannulated screws with washers, carefully positioned to ensure the screw heads were completely recessed and flush with the articular surface, thereby preventing any impingement. The rationale for selecting large, structural allografts from a tissue bank was based on the substantial dimensions and specific anatomical contouring required for effective reconstruction of these defects. Our unit benefits from a consistent supply of bone tissues, updated monthly through a collaboration with the Musculoskeletal Tissue Bank (BTM) of Bologna (Rizzoli Hospital), which ensured the ready availability of appropriate allografts for these procedures. The subscapularis tendon was then re-fixed in its original position with transosseous sutures (as illustrated in Figure 2 and Figure 3). The choice between synthetic grafts, cancellous allografts, or structural femoral-head allografts was based on lesion size and the quality of the residual humeral head bone stock.
- Subscapularis Transposition (McLaughlin Technique) (Randelli Type 1): In Type 1 lesions where the impact injury was not deemed repairable due to chronicity (e.g., 6–8 weeks post-trauma), the subscapularis tendon was transposed into the defect and fixed with transosseous sutures or anchors (as illustrated in Figure 4).
- Reverse Shoulder Arthroplasty (Randelli Type 2 and selected Type 4): For Type 2 lesions, especially in older patients or those with significant chronicity (e.g., 5 months post-trauma), a reverse shoulder prosthesis was implanted (as illustrated in Figure 5). One patient with a Type 4 lesion also received a reverse prosthesis.
- Osteosynthesis (Randelli Type 4): In younger patients with Type 4 multifragmentary fracture dislocations, reduction and internal fixation with plate and screws were performed.

In the 2 patients with repairable type 1 impact injury, in consideration of the time elapsed since the trauma of 3 weeks and their young age, the subscapularis tendon was first detached, then the articular surface was raised, filling the defect with cancellous allograft bone in 1 case and with synthetic bone in the other. Finally, the subscapularis tendon was fixed with transosseous suture in the original position (Figure 2).

In the other 2 patients in whom the impact injury was not repairable due to chronicity (6–8 weeks from the initial trauma), only the subscapularis tendon was transposed inside the injury and fixed transosseous sutures in 1 case and with 2 anchors in the other case (Figure 4).

The remaining 2 patients with type 1 injury 4 weeks after the trauma were treated by detachment of the subscapularis tendon, debridement of the humeral bone bed and the defect was filled with a structural wedge-shaped femoral head allograft fixed with 2 cannulated screws slightly sunk into the articular surface. Subsequently the tendon was sutured back to its original insertion (Figure 3).

In the 2 patients with type 2 injury, a reverse prosthesis was implanted due to the age of the patients being around 60 years and the considerable time that had elapsed since the trauma, in particular for 1 of the 2 approximately 5 months (Figure 5).

The 2 patients treated at 3 weeks for the type 4 lesion were treated with reduction and fixation with plate and screws due to their young ages, 41 and 46 years, respectively.

Postoperatively, all patients were initially treated with an arm brace for 3 weeks for those operated with a reverse prosthesis, while for 4 weeks for those operated with a bone graft or transposition. For post-operative rehabilitation, the patients were treated by phisiotherapists specialized in the upper limb area.

Table 2 summarizes the treatment choices for all patients and the corresponding timing.

All patients were specifically examined for the presence of specific symptoms: pain, muscle strength, stiffness, sensorium, compensation problems. The active ROM and passive ROM (flexion, extra-rotation, intra-rotation) was evaluated. All Range of Motion (ROM) measurements were performed by specialized physiotherapists using standardized goniometric techniques. While a formal blinding protocol for evaluators was not feasible given the retrospective nature of the study, the physiotherapists involved in the assessments were independent of the surgical team, aiming to minimize potential assessment bias. Finally, the general state of health was assessed with the SF-36 score and function using the SDQ (Shoulder Disability Questionnaire) score. Radiographic evaluation was performed at each follow-up with shoulder x-ray with dedicated projections (AP and glenoid profile). A T-test was used for specific comparisons, such as the evaluation of internal rotation loss in patients treated with the McLaughlin technique. Because of the very small and heterogeneous subgroups, we did not calculate confidence intervals or effect sizes, as these would have been statistically unreliable and potentially misleading.

## 3. Results

The average diagnostic delay for our cohort was 6 weeks, with a range spanning from 3 weeks to 5 months, as detailed for each patient in Table 3. Notably, in 4 out of 10 cases (40%), the initial diagnosis of posterior shoulder dislocation was missed. This misdiagnosis was primarily attributed to inadequate radiographic projections or misinterpretation of standard X-rays at the initial presentation, leading to significant delays in appropriate treatment.

All patients were examined and showed painful and locked shoulder in adduction and intrarotation stance.

All were prescribed a CT scan, which always showed chronic lesions.

6 of our 10 patients, following the trauma, only self-medicated. The remaining 4 went to an emergency room, where in 1 case no radiographs were taken, and in the remaining 3 cases, radiographs with unsuitable projections were taken and the patients discharged without the appropriate diagnosis.

All 10 patients included in the study had complete 12-month follow-up data, as patients with incomplete follow-up were excluded during the initial screening process (as detailed in Table 1). Therefore, all reported outcome assessments, including ROM measurements and questionnaire scores, were derived from in-person evaluations, ensuring the validity and consistency of the data.

All 10 patients were willing to undergo the 1-year postoperative follow-up (1 month, 3 months, 6 months and 12 months). 4 patients, given personal contingencies, were unable to undergo all periodic follow-up visits but were still willing to undergo a telephone interview.

At 12 months follow-up, patients treated with reverse prosthesis (n = 3) demonstrated a mean flexion of 155° (SD 2.89°) and mean internal rotation of 57.67° (SD 2.89°). Patients who received bone grafts (n = 4) achieved a mean flexion of 161.25° (SD 6.29°) and mean internal rotation of 69.5° (SD 4.33°). For patients treated with subscapularis transposition (n = 2), the mean flexion was 147.5° (SD 3.54°) and mean internal rotation was 52.5° (SD 3.54°). The patient treated with osteosynthesis (n = 1) had a flexion of 140° and internal rotation of 45°. Individual patient ROM values are detailed in Table 1.

In patients treated with subscapularis transposition (n = 2), a mean internal rotation of 52.5° (SD 3.54) was observed at 12 months. This represents approximately a 10% reduction in internal rotation compared to the mean internal rotation achieved in patients treated with bone grafts (mean 69.5°, SD 4.33°).

In the area of postoperative complications, we recorded one patient with a type 4 fracture who at about 6 months began to develop necrosis picture with subsequent sub-total resorption of the humeral head. This patient was subsequently performed surgery to remove synthetic means, but given the evolution of the pathological picture, implantation of prosthesis was suggested, to which he preferred to date to postpone.

Table 3 shows all the functional results in terms of ROM in flexion and internal rotation, as well as the evaluation of the SF-36 and the SQ-score for each patient analyzed.

At 12 months, functional outcomes showed similar patterns across treatment groups. Mean SDQ scores were 5 (SD 1) for reverse prosthesis (n = 3), 2.5 (SD 1.29) for bone grafting (n = 4), and 5.5 (SD 0.71) for subscapularis transposition (n = 2), while the patient treated with osteosynthesis scored 8. No cases of biceps pain were reported at final follow-up, and all individual values are summarized in Table 1.

For the SF-36 Physical Functioning domain, bone graft patients (n = 4) showed the highest mean score (79.5, SD 4.33), followed by subscapularis transposition (67.5, SD 3.54; n = 2) and reverse prosthesis (66, SD 5.29; n = 3). The osteosynthesis case recorded a score of 50. Detailed patient-level data are provided in Table 1.

## 4. Discussion

The clinical results obtained with the original or modified McLaughlin procedures in patients with locked posterior shoulder dislocation were favorable and comparable with those of other published studies [7,13,14].

McLaughlin called posterior shoulder dislocation a “diagnostic trap” [11] because of the high percentage of cases missed on initial presentation. This was also the case in our study, with a significant delay between dislocation and reduction (6 weeks on average). However, even with this treatment delay in a traumatic intra-articular pathology, the clinical outcomes were still favorable, with significant improvements in all clinical variables.

The management of chronic posterior shoulder dislocations remains a significant challenge, as highlighted by Loebenberg and Cuomo (2000), who emphasize the importance of early recognition and careful patient selection due to the guarded outcomes often associated with these injuries [14].

Results demonstrate that the surgical treatment of chronic locked posterior shoulder dislocation, guided by an individualized approach based on the Randelli classification, leads to favorable outcomes in this complex patient population. For instance, our patients treated with bone grafts achieved a mean flexion of 161.25° and internal rotation of 69.5°, with an average SDQ score of 2.5. These outcomes are comparable to, or even exceed, those reported in other series. For example, Ippolito et al. (2021), in a study comparing open and arthroscopic McLaughlin procedures, reported mean Constant scores of 81.3° for open and 80.25° for arthroscopic techniques, with SST scores of 10.8° and 11.5°, respectively [13]. Our SF-36 Physical Functioning scores, averaging 79.5° for bone grafts and 66° for reverse prosthesis, align well with the functional improvements noted in similar studies. The initially superior ROM observed in reverse prosthesis patients likely reflects immediate joint congruity and stability provided by the implant, whereas graft-based reconstructions require biological incorporation before full recovery is achieved.

Furthermore, all patients treated with subscapularis tendon transposition experienced a slight loss of internal rotation and increased pain over the long head of the biceps. This observation aligns with findings in the literature, where subscapularis transposition, while effective in filling the bone defect and restoring stability, can lead to some degree of internal rotation restriction due to the medialization of the tendon’s insertion [12,15]. Besnard et al. (2019) specifically mention the theoretical risk of reduced internal rotation short-term as a disadvantage of the arthroscopic McLaughlin technique [12]. Given these functional trade-offs and the potential for residual pain, we advocate for a cautious approach. Therefore, taking this data into account, we believe it is appropriate to resort to subscapularis transpositions primarily when stability cannot be reliably achieved by less aggressive methods, such as bone grafting, which in our series demonstrated superior internal rotation and overall functional scores. This strategy aims to optimize functional outcomes and minimize postoperative discomfort, reserving techniques with potential functional limitations for situations where they are indispensable for joint stability

Patients undergoing reverse total shoulder arthroplasty show good functional recovery (mean flexion 155°, mean internal rotation 57.67°), consistent with the literature supporting arthroplasty for severe defects in older patients or those with extensive bone loss [14]. This is further exemplified by the case report by Salomon et al. (2021), which, despite being a single case, underscores how unrecognized PSDs with significant bone loss and rotator cuff tear ultimately necessitated reverse total shoulder arthroplasty, leading to a successful return to recreational cycling [3]. The reported SF-36 Physical Functioning score of 73° and SDQ score of 4 in that case align well with the outcomes observed in our cohort for similar interventions.

The arthroscopic McLaughlin procedure, as described by Besnard et al. (2019) and Bernholt et al. (2020), emphasizes effective stabilization and reduced morbidity [12,15]. Our results for subscapularis transposition (mean flexion 147.5°, internal rotation 52.5°) reflect a functional recovery consistent with the expectations for this technique, albeit with a noted internal rotation loss. These comparisons underscore the effectiveness of our tailored treatment strategies in achieving functional recovery and pain relief, even in this challenging cohort, and reinforce the critical role of individualized treatment based on lesion characteristics and patient needs, as also discussed by Loebenberg and Cuomo (2000) [14]. Although statistically detectable, a 10% internal rotation deficit corresponds to a small functional difference that did not significantly affect daily living activities in our cohort.

Our study observed one case of avascular necrosis (AVN), representing an incidence of 9% within our small cohort. This incidence, while seemingly higher than some reported figures (e.g., 3.5% in certain series), must be interpreted cautiously given our limited sample size. The patient in question presented with a Type 4 multifragmentary fracture dislocation and experienced a diagnostic delay of 3 weeks, followed by surgical reduction. Factors contributing to the development of AVN in chronic posterior fracture dislocations are multifactorial, often including the severity of the initial trauma, the extent of vascular disruption to the humeral head, and crucially, the duration of the unreduced dislocation [14]. In our case, the multifragmentary nature of the Type 4 lesion likely compromised the vascular supply to the humeral head, and despite surgical reduction at 3 weeks, the initial insult combined with the inherent fragility of the vascularity in such complex fractures predisposed to AVN. Loebenberg and Cuomo (2000) highlight that the longer the delay between occurrence and diagnosis, the more limited the treatment options become and the more guarded the prognosis, often leading to complications like AVN [14]. While the patient ultimately achieved a good clinical outcome with acceptable function and no pain, not requiring immediate shoulder arthroplasty, the development of AVN underscores the critical importance of early diagnosis and prompt, appropriate intervention to preserve humeral head viability, especially in complex fracture dislocations.

The limitations of this study include its retrospective nature, small number of pa-tients (which reflects the rarity of this pathology), the fact that it was conducted in only two centers and, in some cases, relatively short follow-up. As a result, this study suffers from inadequate case representation and case selective bias. In addition, the limited sample size precluded robust statistical comparisons between treatment subgroups and did not allow for a meaningful estimation of effect sizes or confidence intervals. However, this is a very rare pathology so most of the series in the literature have the same weaknesses.

A further limitation stems from the small and heterogeneous nature of our patient cohort, which, while typical for studies on such a rare pathology, precluded robust statistical comparisons between different surgical subgroups. The highly individualized treatment approaches, tailored to specific lesion characteristics and patient profiles, resulted in very small group sizes, making formal comparative statistical analyses unreliable and potentially misleading.

## 5. Conclusions

Early diagnosis and treatment of PSDs and an associated McLaughlin’s lesion are therefore crucial and can prevent the adverse sequelae associated with this condition, such as degenerative shoulder disease, increased bone defects (e.g., McLaughlin’s lesion extension and posterior glenoid rim fractures), chronic rotator cuff tears, and avascular necrosis of the humeral head. Therefore, it is of paramount importance to perform a scrupulous diagnostic procedure through iconography performed with the correct projections and careful examination of the same iconography and clinical evaluation.

Osteosyntheses should be considered only in extremely exceptional cases because of the high risk of necrotic evolution. Based on our observation of one case of avascular necrosis following osteosynthesis in a Type 4 multifragmentary fracture dislocation, we suggest that osteosynthesis in such complex scenarios requires extremely careful patient selection and consideration, particularly given the inherent risk of vascular compromise and necrotic evolution. This group of patients with chronic posterior shoulder dislocation, treated with replacement, grafting, transposition, ostheosynthesis and prosthetic replacement, had good clinical results, even when surgical reduction was significantly delayed.

Considering the slight residual internal rotation deficit and the increase in pain on the long head of the biceps, we believe it is appropriate to resort to subscapularis transpositions only when stability cannot be ensured by other equally aggressive surgical methods.

## Figures and Tables

**Table 2:** Overview of treatment allocation and timing according to Randelli lesion type.

PATIENTS	AGE	TIMING	SURGERY
6 Type 1	38.5 (25–47)	2 patients 3 weeks 2 patients 6–8 weeks 2 patients 4 weeks	2 wads 2 transpositions 2 grafts
2 Type 2	54.25 (49–58)	2 patients 8 weeks	2 reverse
2 Type 4	43.5 (41–46)	3 weeks	1 osteosynthesis 1 reverse prosthesis

**Table 3:** Individual clinical outcomes for the ten patients at 12-month follow-up.

Patient ID	1	2	3	4	5	6	7	8	9	10
Age (years)	25	30	40	47	35	38	49	58	41	46
Gender	M	M	M	M	M	M	M	M	M	M
Side	Dx	Dx	Dx	Sx	Dx	Dx	Dx	Sx	Dx	Sx
Randelli Type	Type 1	Type 1	Type 1	Type 1	Type 1	Type 1	Type 2	Type 2	Type 4	Type 4
Diagnostic Delay	3 weeks	3 weeks	4 weeks	4 weeks	6 weeks	8 weeks	8 weeks	5 months	3 weeks	3 weeks
Surgical Procedure	Cancellous allograft	Synthetic bone graft	Femoral head allograft + screws	Femoral head allograft + screws	Subscapularis transposition	Subscapularis transposition	Reverse prosthesis	Reverse prosthesis	Osteosynthesis (plate + screws)	Reverse prosthesis
ROM Flexion (12 m)	170°	165°	160°	155°	150°	145°	160°	155°	140°	150°
ROM Flexion (3 m)	155°	150°	140°	140°	135°	140°	155°	150°	130°	140°
SDQ Score (12 m)	1	2	3	4	5	6	4	5	8	6
SF-36 Physical Functioning (12 m)	85	80	78	75	70	65	70	68	50	60
Complications	None	None	None	None	Minor IR loss, increased pain	Minor IR loss, increased pain	None	None	Avascular necrosis (no further surgery yet)	None

## Data Availability

The data that support the findings of this study are available from the corresponding Author upon reasonable request.
